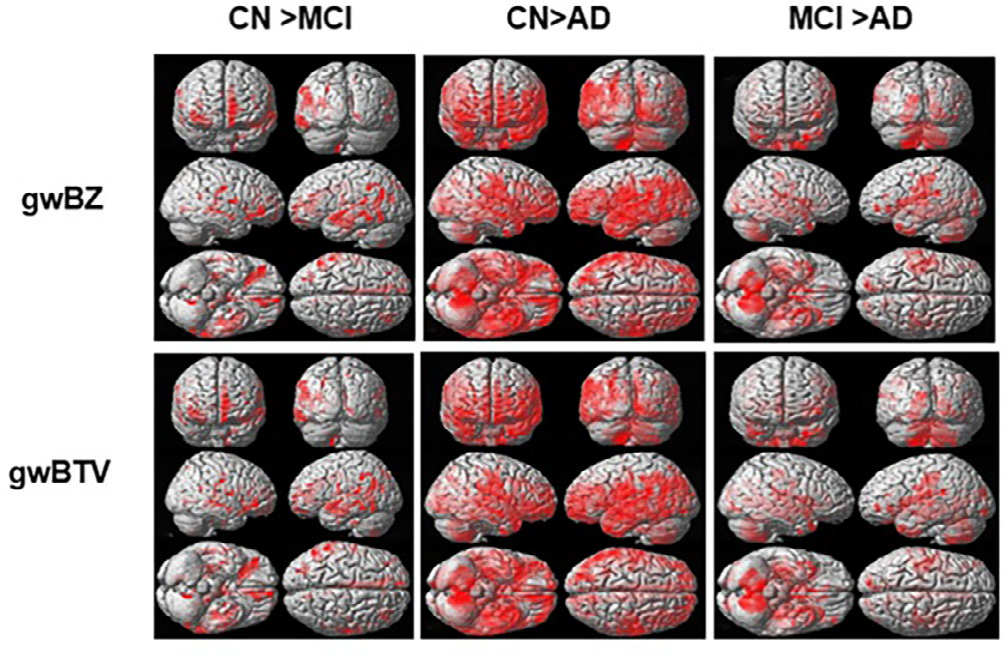# Gray‐White Matter Boundary Z‐Score and Its Volume as Imaging Biomarkers of Alzheimer’s Disease

**DOI:** 10.1002/alz.085486

**Published:** 2025-01-09

**Authors:** Geon‐Ho Jahng, Yunan Tian, Jang‐Hoon Oh, Hak Young Rhee, Soonchan Park, Chang‐Woo Ryu

**Affiliations:** ^1^ Kyung Hee University Hospital at Gangdong, Seoul Korea, Republic of (South); ^2^ Kyung Hee University College of Medicine, Seoul Korea, Republic of (South); ^3^ Kyung Hee University Hospital, Seoul Korea, Republic of (South)

## Abstract

**Background:**

Alzheimer's disease (AD) presents typically gray matter atrophy and white matter abnormalities in neuroimaging, suggesting that the gray‐white matter boundary could be altered in individuals with AD. The purpose of this study was to explore differences in gray‐white matter boundary Z‐score (gwBZ) and its tissue volume (gwBTV) between patients with AD, amnestic mild cognitive impairment (MCI), and cognitively normal (CN) elderly participants.

**Method:**

Three‐dimensional T1‐weight images of a total of 227 participants were prospectively obtained to map gwBZ and gwBTV on images using a 3‐T MR system. Statistical analyses of gwBZ and gwBTV maps were performed using the full factorial one‐way analysis of covariance (ANCOVA) to compare among the three groups and the multiple regression analysis to assess the association between each map and MMSE score with both age and TIV as covariates.

**Result:**

This study included 62 CN participants (71.8 ± 4.8 years, 20 males, 42 females), 72 MCI participants (72.6 ± 5.1 years, 23 males, 49 females), and 93 AD participants (73.6 ± 7.7 years, 22 males, 71 females). It was found that gwBZ and gwBTV were lower in AD than in CN or MCI and lower in MCI than in CN. The AD group had lower gwBZ and gwBTV than the CN and MCI groups. MMSE showed positive correlations with gwBZ and gwBTV whereas age showed negative correlations with gwBZ and gwBTV. The combination of gwBZ or gwBTV with MMSE had a high accuracy in classifying AD from CN in the hippocampus with an area under curve (AUC) value of 0.972 for both.

**Conclusion:**

gwBZ and gwBTV were reduced in AD. They were correlated with cognitive function and age. Moreover, gwBZ or gwBTV combined with K‐MMSE had a high accuracy in differentiating AD from CN in the hippocampus. These findings suggest that evaluating gwBZ and gwBTV in the AD brain could be a useful tool for monitoring AD progression and diagnosis.